# Simulation-based Training for Pelvic and Breast Physical Examination: Effect on the Anxiety and Self-confidence of Medical Students

**DOI:** 10.1055/s-0040-1718433

**Published:** 2020-11-30

**Authors:** Talles Dias Orsi, Ana Lucia Ribeiro Valadares, Paula Miranda Esteves Orsi, Isabella Miranda Esteves Orsi, Alexandre Sampaio Moura

**Affiliations:** 1 Universidade José do Rosário Vellano, Alfenas, MG, Brazil; 2 Universidade Estadual de Campinas, Campinas, SP, Brazil

**Keywords:** vaginal examination, medical students, simulation-based training, anxiety, satisfaction, exame ginecológico, estudantes de medicina, treinamento por simulação, ansiedade, satisfação

## Abstract

**Objective** To evaluate factors associated with anxiety and the effect of simulation-based training (SBT) on student anxiety, self-confidence and learning satisfaction in relation to pelvic and breast examination.

**Methods** A longitudinal study was conducted with 4 ^th^ year medical students at the Universidade José do Rosário Vellano. A 12-item, self-report questionnaire on student anxiety at performing gynecological examinations was applied before and after SBT, with answers being given on a Likert-type scale. After training, the self-confidence levels and satisfaction of the students related to the learning process were also evaluated.

**Results** Eighty students with a mean age of 24.1 ± 4.2 years were included in the study. Of these, 62.5% were women. Pre-SBT evaluation showed that students were more anxious at performing a pelvic examination than a breast examination (2.4 ± 1.0 versus 1.7 ± 0.8, respectively; *p* < 0.001). The primary reason for anxiety regarding both pelvic and breast examination was fear of hurting the patient. SBT significantly reduced student anxiety (2.0 ± 0.8 versus 1.5 ± 0.5, respectively; *p* < 0.001). The satisfaction and self-confidence of the students were found to be high (6.8 ± 0.3 and 6.0 ± 0.9, respectively), with no difference between genders.

**Conclusion** The use of SBT in teaching students to perform pelvic and breast examinations resulted in reduced anxiety and increased self-confidence in a group of medical students of both genders, with high levels of satisfaction in relation to the training.

## Resumo

**Objetivo** Avaliar os fatores relacionados à ansiedade e ao efeito do treinamento baseado em simulação (TBS) na ansiedade, autoconfiança e satisfação do estudante em relação ao aprendizado do exame pélvico e de mamas.

**Métodos** Estudo longitudinal com alunos do quarto ano de medicina da Universidade José do Rosário Vellano, utilizando questionário autorrespondido com 12 itens em escala tipo Likert sobre a ansiedade em relação ao exame ginecológico, pré e pós-TBS. Após o treinamento, avaliou-se também o grau de autoconfiança e satisfação com o processo de aprendizado.

**Resultados** Foram incluído **s** 80 alunos com média etária de 24,1 ± 4,2 anos, dos quais 62,5% eram mulheres. Na avaliação pré-treinamento, a ansiedade dos alunos em relação ao exame pélvico foi maior do que a ansiedade em relação ao exame das mamas (2,4 ± 1,0 *versus* 1,7 ± 0,8, respectivamente; *p* < 0,001). O principal motivo de ansiedade em relação tanto ao exame pélvico quanto ao de mamas foi o receio de machucar a paciente. O TBS reduziu significativamente a ansiedade dos alunos (2,0 ± 0,8 versus 1,5 ± 0,5, respectivamente; *p* < 0,001). A satisfação e autoconfiança dos estudantes foram elevadas (6,8 ± 0,3 e 6,0 ± 0,9, respectivamente), sem diferença entre gêneros.

**Conclusão** O uso de TBS no ensino de exame pélvico e mamário resultou em uma diminuição na ansiedade e elevada autoconfiança em um grupo de estudantes de medicina, de ambos os gêneros, e bons níveis de satisfação em relação ao treinamento.

## Introduction

A general practitioner's training involves developing the skills required to perform pelvic and breast examinations, which are vital for the detection of pathologies of the breast, of the pelvic cavity, of the vagina and of the vulva during physical examination. 1 2 Nevertheless, undergraduate training aimed at teaching students to perform gynecological examinations can cause discomfort to patients and embarrassment among students. 3 Therefore, to minimize these problems, the use of mannequins and models for pelvic examination training has been considered a practical and effective teaching strategy in the initial stages of student training. 4 5

The use of gynecological teaching models is not recent. Indeed, small wax or wooden figures have already been used for several centuries to demonstrate reproductive processes, contraceptive techniques and gynecological conditions. 6 Nevertheless, these models and mannequins used for simulation in medical training have advanced considerably over recent decades, now ranging from simple objects or devices to the representation of a body part or even an entire patient. Consequently, these devices now allow a variety of applications, ranging from their use in teaching a specific skill to the simulation of a wide range of routine clinical situations. 2 6

In a meta-analysis that included 22 studies and 2,036 students, Dilaveri et al 2 showed that mannequin-based simulation involving technological content, conducted to teach students to perform breast and pelvic physical examinations, resulted in significant improvements in student learning compared with no intervention. 2 The magnitude of the beneficial effects of this learning strategy differed from study to study; however, all the studies reported some benefit, leading to the conclusion that, in general, this type of training is indeed useful.

The advantages of simulation-based training (SBT) were also shown in a study conducted by Bouet et al 7 in France in which pelvic examination mannequins were used to train medical students. The level of perceived technical difficulty diminished following training using this teaching method. 7 Furthermore, the benefits of this intervention became evident when a questionnaire was used to rate self-evaluation and satisfaction at the beginning and end of a gynecological simulation training session. 8 In a study conducted by Pugh et al 9 in the United States of America (USA), in which students initially watched a video on pelvic examination and later participated in training with the use of appropriate mannequins before performing pelvic examinations on real patients, results showed significant improvements in student anxiety with respect to all aspects of the examination. 9

Concerning breast examination, a study conducted in the USA found that medical students who learned to perform a clinical examination using a breast palpation simulator performed as well as or better than those who learned on standardized patients. Nevertheless, an analysis performed on a subgroup showed that the benefit was limited to students with less clinical experience. No statistically significant differences in self-confidence were found between the two groups. 10 Another study conducted in the USA attempted to identify sources of student anxiety when learning to perform clinical breast examination and to evaluate the effects on anxiety of learning using simulated breast models. Fear of missing a breast lesion and the intimate/personal nature of the examination accounted for 73.8% of causes of student anxiety. Anxiety levels in students with respect to performing clinical examinations decreased significantly following training with SBT. 11

Most international studies have shown that following SBT students experience a significant reduction in their initial anxiety at having to perform pelvic or breast examinations. 9 11 Nevertheless, few studies have been conducted in Brazil to evaluate the use of SBT in teaching students to perform pelvic and breast examination. Although cultural and financial differences may limit the extrapolation of findings from international studies, it is clear that low-fidelity simulation and examination mannequins for pelvic and breast examination represent a useful training approach. Bouet et al 7 reported that this low-cost strategy is used in France to prepare students to perform gynecological examinations. In France, as in Brazil, standardized patients are not used in simulation training in gynecology for cultural reasons. This practice is certainly different from those of Anglo-Saxon countries where standardized patients are common practice. 8

In view of the aforementioned reasons, the objective of the present study was to evaluate the factors associated with anxiety and the effect of SBT on anxiety, self-confidence and student satisfaction in relation to pelvic and breast examination.

## Methods

A longitudinal, observational study was conducted with students enrolled in the 7 ^th^ semester of medical school at the Universidade José do Rosário Vellano in Alfenas, Minas Gerais, Brazil. It was during this semester (between March and July 2018) that the students participated in the Obstetrics and Gynecology (Ob/Gyn) clerkship.

As part of the Ob/Gyn clerkship program, the students attended a theoretical class on Ob/Gyn anamnesis and watched a video showing, step by step, how to perform pelvic and breast examinations. The students who agreed to participate in the study then completed a sociodemographic questionnaire and a form containing questions to be rated on a 7-point Likert-type scale regarding their self-perceived anxiety in relation to performing pelvic and breast examinations. This questionnaire was adapted from the Fear of Pelvic Examination Scale (F-PEXS) proposed by Siwe et al. 12 The F-PEXS was chosen because it has shown outstanding reliability and the right construct validity scale in a previous study. Although F-PEXS has not yet been validated for use in Brazilian Portuguese language, the items are quite straightforward, and a pilot study with a group of 28 medical students was conducted to make semantic adjustments.

After completing these instruments, the students participated in the first SBT session. During that session, the students were divided into three groups, with each group being assigned to one mannequin. Two types of mannequins were used to teach the students how to perform a pelvic examination: a pelvic simulator for gynecological examination and a pelvic simulator for cervical examination (dilatation). Each student underwent individual training, supervised by the professor assigned to that particular group. They were taught how to perform a speculum examination, how to collect cervical cytology specimens, and how to perform a digital vaginal examination to evaluate cervical dilatation. The following day, the students participated in the second SBT session, in which they were taught to perform a clinical breast examination, including palpation for lumps, evaluation of nipple discharge and palpation of axillary and supraclavicular lymph nodes on a mannequin. Two types of mannequins were used at the second session, one representing the female thorax and the other composed of single breasts attached to a base. A professor assigned to each group also supervised this training. Each session lasted 2 hours and, after completion, the students answered a second questionnaire containing questions on anxiety regarding pelvic and breast examination. These questions were identical to those contained in the form applied at baseline but also included the questions on the Student Satisfaction and Self-Confidence in Learning Scale validated by Almeida et al 5 for Brazilian Portuguese.

### Statistical Analysis

The scores obtained from the Likert-type scale were calculated by adding the ratings for each answer within the domain of interest and then dividing the sum by the number of questions in that domain. Ratings ranged from 1 to 7, in which 1 meant “I completely disagree” with the statement and 7 meant “I completely agree” with the statement. Students scoring a mean of 5 or higher in the anxiety domain were considered to be extremely anxious.

The Student *t* -test for paired samples was used to compare mean anxiety scores measured prior to and following SBT. The Pearson correlation coefficient was used to evaluate the association between pre- and post-SBT anxiety scores and to evaluate the association between satisfaction and self-confidence, satisfaction and anxiety, and self-confidence and anxiety. The Student *t* -test for independent samples was used to evaluate whether the scores assessed for anxiety, satisfaction and self-confidence differed between female and male students. Cronbach's α was used to assess the internal consistency of the questions in each of the three domains evaluated.

## Results

A total of 80 students were included in the study. Of these, 62.5% were women and the mean age of the participants was 24.1 ± 4.2 years. Most stated that they had already initiated their sexual life and only two of the students reported being homosexual. Only 12 students reported having had previous experience in women's health, either as an intern or when they participated in an academic league ( Table 1 ).

**Table 1:** Sociodemographic and behavioral characteristics of the students

	Frequency	
Sociodemographic and behavioral characteristics	n *	%
Type of secondary education		
Public	9	11.4
Private	69	87.3
Public and Private	1	1.3
Does the student perform any paid work?		
Yes	1	1.2
No	79	98.8
Monthly family income		
≤ R$ 5,000.00	6	7.7
Between R$ 5,001.00 and R$ 12,000.00	27	34.6
> R$ 12,000.00	45	57.7
Is currently participating in or has participated in an extension program or academic league in gynecology?		
Yes	5	6.2
No	75	93.8
Is currently participating or has participated in a practical work experience in gynecology?		
Yes	7	8.8
No	73	91.2
Has already initiated his/her sexual life?		
Yes	77	96.3
No	3	3.8
At what age did you first have sexual intercourse? (For the students who have already initiated their sexual life)		
13–15 years	19	26.4
16–18 years	39	54.1
> 18 years	14	19.5
Sexual orientation		
Heterosexual	76	97.4
Homosexual	2	2.6
Have you ever been submitted to a digital rectal examination?		
Yes	5	6.2
No	75	93.8
Have you ever been submitted to a gynecological examination? (Only for female students)		
Yes	48	96.0
No	2	4.0

* Although the overall database consisted of 80 students, the number of students does not always add up to 80 for all the variables analyzed due to missing data in some cases.

The internal consistency of the questions measured using Cronbach's α showed good reliability and good internal consistency in the different domains.

Baseline overall anxiety was low (2.04 ± 0.80) and, in general, the students' anxiety in relation to pelvic examination was found to be greater than their anxiety regarding breast examination (2.38 ± 0.98 versus 1.70 ± 0.79, respectively; *p* < 0.001). When anxiety levels were compared between the groups of male and female students, no significant differences were found, with the following mean baseline scores being recorded for the two groups, respectively: anxiety regarding pelvic examination (2.33 ± 1.01 versus 2.44 versus ± 1.00; *p* = 0.649), anxiety regarding breast examination (1.84 ± 0.88 versus 1.63 ± 0.74; *p* = 0.317), and anxiety regarding both pelvic and breast examinations (2.11 ± 0.90 versus 2.00 ± 0.75; *p* = 0.594).

The causes of anxiety in relation to pelvic examination were never having seen a pelvic examination (24.0%), not knowing how to perform a pelvic examination, fear of hurting the patient or of being clumsy (44.0%), fear of the patient feeling uncomfortable (20.0%), and the students themselves feeling uncomfortable with the examination (8.0%). The causes of anxiety in relation to breast examination were fear of hurting the patient or of being clumsy (33.3%), fear of making the patient feel uncomfortable (33.3%), feeling uncomfortable (22.2%) and never having seen the exam performed (11.2%).

When stratified according to gender, the main reason for anxiety reported by the female students was fear of hurting the patient, both in relation to the pelvic examination and to the breast examination. The main reason for anxiety reported by the male students was never having performed the exam previously and fear of making the patient feel uncomfortable.

The anxiety scores in relation to the overall gynecological examination decreased significantly from 2.04 ± 0.80 at baseline to 1.46 ± 0.50 following SBT ( *p* < 0.001). Regarding anxiety in relation to pelvic examination specifically, the mean score of 2.40 ± 1.00 prior to SBT decreased to 1.64 ± 0.68 following the intervention ( *p* < 0.001), while the mean score of 1.70 ± 0.79 for anxiety regarding breast examination prior to SBT fell to 1.33 ± 0.46 following SBT ( *p* < 0.001) ( Table 2 ).

**Table 2:** Comparison of scores for anxiety with respect to the gynecological examination between baseline evaluation and the evaluation performed following simulation-based training

Anxiety Scores		Descriptive Measures				
Phase	n	Minimum	Maximum	Mean	SD	*p-value* *
Pelvic examination						
Baseline	80	1.00	5.17	2.40	1.00	
Post-SBT	80	1.00	3.33	1.64	0.68	< 0.001
Baseline - Post-SBT				0.76	0.82	
Breast examination						
Baseline	72	1.00	4.67	1.70	0.79	
Post-SBT	72	1.00	3.00	1.33	0.46	< 0.001
Baseline - Post-SBT				0.37	0.64	
Pelvic and breast examination						
Baseline	72	1.00	4.92	2.04	0.80	
Post-SBT	72	1.00	3.00	1.46	0.51	p < 0.001
Baseline - Post-SBT				0.58	0.64	

Abbreviations: SBT, simulation-based training; SD, standard deviation.

* Student's *t* -test for paired samples. The number of students differs from the total sample of 80 students due to missing data for some of the variables.

The analysis of the correlation between the anxiety scores evaluated prior to and following SBT is shown in Table 3 .

**Table 3:** Analysis of the correlation between the anxiety scores evaluated prior to and following simulation-based training ( *n* = 80)

Scores	r	*p-value* *
Anxiety regarding pelvic examination: baseline versus post-SBT	0.53	< 0.001
Anxiety regarding breast examination: baseline versus post-SBT	0.60	< 0.001
Anxiety regarding pelvic and breast examination: baseline versus post-SBT	0.61	< 0.001

Abbreviation: SBT, simulation-based training.

*r* = Pearson's correlation.

* Student's *t* -test for paired samples.

The levels of anxiety observed prior to SBT decreased after SBT in both genders. As at baseline, following SBT there was no difference in the level of anxiety between genders, either for the overall gynecological examination (mean 1.53 ± 0.62 versus 1.47 ± 0.52 for males and females, respectively; *p* = 0.67) or for the pelvic examination (1.66 ± 0.74 versus 1.63 ± 0.65, respectively; *p* = 0.86) or breast examination (1.41 ± 0.56 versus 1.32 ± 0.47, respectively; *p* = 0.47).

When the group of students with previous experience in women's health (resulting from their participation in academic leagues or extension activities) was compared with the other students. Those with prior experience were found to be less anxious with respect to the overall clinical examination prior to SBT (1.55 ± 0.45 versus 2.12 ± 0.82, respectively; *p* = 0.004), with none of the students being classified as extremely anxious. Following SBT, the difference between the group with previous experience and the other students in relation to the overall clinical examination was no longer statistically significant (1.32 ± 0.27 versus 1.48 ± 0.54, respectively; *p* = 0.14).

Student satisfaction was very high (mean 6.85 ± 0.39) and there was no statistically significant difference between the female and male students. Self-confidence was also high (5.97 ± 0.88) following SBT, with no difference between genders. Satisfaction with SBT was similar between the group of students with previous experience in women's healthcare and the remaining students (6.88 ± 0.23 versus 6.86 ± 0.41, respectively; *p* = 0.84); however, self-confidence, although high in both groups, was higher in the group with previous experience (6.41 ± 0.67 versus 5.89 ± 0.89, respectively, *p* = 0.03).

No statistically significant correlation was found between the degree of student anxiety and the student's satisfaction with the learning experience (r = -0.04; *p* = 0.771) or between the degree of student anxiety and his/her self-confidence (r = -0.07; *p* = 0.561) ( Table 4 ).

**Table 4:** Analysis of the correlation between anxiety scores prior to simulation-based training and scores for learning-related satisfaction and self-confidence in learning ( *n* = 80)

Scores	Satisfaction (p-value) *	Self-Confidence (p-value) *
Anxiety regarding pelvic examination	−0.03 (0.799)	−0.10 (0.409)
Anxiety regarding breast examination	−0.04 (0.763)	−0.02 (0.867)
Anxiety regarding pelvic and breast examination	−0.04 (0.771)	−0.07 (0.561)

Database: 80 students.

* Pearson correlation analysis. Correlation analysis between anxiety regarding pelvic examination and anxiety regarding breast examination (r = 0.64; *p* < 0.001).

## Discussion

Simulation-based training effectively reduced anxiety in medical students of both genders, both in relation to pelvic and breast examinations. Furthermore, after training, scores for satisfaction and self-confidence were high. Therefore, in the present study, the effect of SBT in reducing anxiety in relation to pelvic and breast examination is in agreement with reports from earlier studies. 7 8 9 11

The present study showed, however, that insofar as the effects of SBT were concerned, there was no statistically significant difference between male and female students, with a decrease in anxiety in both genders. Nevertheless, no statistically significant correlation was found between anxiety level and learning-related satisfaction or between anxiety level and self-confidence.

At the baseline evaluation, the students experienced less anxiety in relation to breast examination than in relation to pelvic examination. Hugon-Rodin et al 8 suggested that the finding of less discomfort with breast examination compared with pelvic examination could be explained by the less intimate and more discrete nature of that examination. Those authors did not mention any differences between genders.

In the present study, the main cause of student anxiety with respect to pelvic examination was fear of hurting the patient (44%), followed by never having seen a pelvic examination performed (24%). When the replies were analyzed according to gender, it was found that the female students mentioned fear of hurting the patient as the principal cause of their anxiety, whereas the men emphasized never having performed the exam. Pugh et al 9 investigated the main source of student anxiety when learning to perform a clinical pelvic examination on women and found that fear of causing harm or pain was the most common answer given by medical students (49.7%). This was followed by the intimate/personal nature of the exam (25.7%). Conversely, a study conducted in France, in agreement with a study conducted in Australia, showed that the majority of students (62%) felt embarrassed because they had never performed a gynecological examination. 8 13 The Australian study also identified embarrassment as the main cause (42%) followed by insecurity at performing a pelvic examination.

In relation to the breast examination, fear of hurting the patient and fear of the patient feeling uncomfortable were the main causes of anxiety prior to SBT. Conversely, according to a study conducted by Pugh et al 11 in a university in the USA, fear of missing a breast lesion and the intimate/personal nature of the exam were responsible for 73.8% of the anxiety experienced prior to training.

In the present study, the students' satisfaction and self-confidence were high following SBT, with no statistically significant differences between genders. These results are consistent with other studies published in the literature in relation to SBT in gynecology, in which satisfaction rates for students are reported as being > 90% following training. 8

Students who had participated in gynecology leagues or who had extracurricular practical experience in gynecology had less overall anxiety prior to SBT in relation to the group with no previous experience. It is interesting to note, however, that there was no statistically significant difference in overall anxiety between these two groups following SBT. To the best of our knowledge, there are no studies in the literature that have specifically evaluated this subgroup of students with greater prior exposure to the subject matter.

The limitations of the present study include the fact that measures of anxiety and confidence were based on students' self-perception. According to Deladisma et al, 14 since measures of anxiety and confidence are subjective and self-reported, they may not correlate with actual levels. In addition, the F-PEXS has not yet been validated for use in Brazil. However, the questions contained in this scale are straightforward and easily understandable, and the authors performed rigorous adaptation procedures in a group of students with similar characteristics to those of the study participants until the final version was considered adequate, that is, until the participants reported no more doubts or difficulties in answering the questions. Another limitation refers to the fact that the study was conducted in a single institution; therefore, the results cannot be extrapolated to other settings. In addition, the sample size was small and predominantly female. Although this sample size was sufficient to allow a reduction in anxiety following SBT to be detected, it may have been insufficient to detect differences between genders. The finding of low overall anxiety at baseline may also have limited the capacity of the study to detect certain differences in the subanalyses. This low baseline level of anxiety could be related to the fact that the students had already watched an educational video and a lecture on how to perform a gynecological examination. Nevertheless, statistically significant differences were found between the analyses conducted prior to and following SBT, highlighting the importance of this strategy.

## Conclusion

The main cause of anxiety in relation to pelvic and breast examination was the fear of hurting the patient. In our opinion, it is vital to deal with student anxiety in the initial phases of learning, since this could affect their performance in clinical practice. Therefore, knowledge on the factors associated with anxiety is important to improve the way in which they are managed. The use of SBT in a group of 7 ^th^ semester medical students showed a reduction in the anxiety scores with respect to pelvic and breast examinations, with a high rate of satisfaction and self-confidence in relation to their ability to perform a gynecological examination. Future studies with larger sample sizes and conducted in different medical schools will provide more data with which to extend the present analysis.

## References

[1] Martins A PV Visões do feminino: a medicina da mulher nos séculos XIX e XX Rio de Janeiro Editora Fiocruz 2004

[2] Dilaveri C A Szostek J H Wang A T Cook D A Simulation training for breast and pelvic physical examination: a systematic review and meta-analysis BJOG 2013 120 10 1171 1182 10.1111/1471-0528.12289 23750657

[3] Rio S MP Trivellato I M Caldeira N M Araujo S F Rezende D F [Experience of women attended by medical students in gynecological care] Rev Bras Educ Med 2013 37 04 492 500 10.1590/S0100-55022013000400004

[4] Okuda Y Bryson E O DeMaria S Jr Jacobson L Quinones J Shen B Levine A I The utility of simulation in medical education: what is the evidence? Mt Sinai J Med 2009 76 04 330 343 10.1002/msj.20127 19642147

[5] Almeida R GS Mazzo A Martins J CA Baptista R CN Girão F B Mendes I AC Validation to Portuguese of the Scale of Student Satisfaction and Self-Confidence in Learning Rev Lat Am Enfermagem 2015 23 06 1007 1013 10.1590/0104-1169.0472.2643 26625990 PMC4663999

[6] Gardner R Raemer D B Simulation in obstetrics and gynecology Obstet Gynecol Clin North Am 2008 35 01 97 127, ix 10.1016/j.ogc.2007.12.008 18319131

[7] Bouet P E Jeanneteau P Legendre G Hachem H E Richard I Granry J-C [Training of medical students for pelvic examination: Benefits of teaching on anatomic models] J Gynecol Obstet Biol Reprod (Paris) 2016 45 07 679 684 10.1016/j.jgyn.2015.09.006 26996238

[8] Hugon-Rodin J Sonigo C Drummond D Grynberg M Rodin T Plu-Bureau G Tesniere A [Learning the gynecological examination on low-fidelity simulation: Impact on the feelings of medical students] Gynécol Obstét Fertil Sénol 2017 45 05 291 298 10.1016/j.gofs.2017.03.011 28461236

[9] Pugh C M Obadina E T Aidoo K A Fear of causing harm: use of mannequin-based simulation to decrease student anxiety prior to interacting with female teaching associates Teach Learn Med 2009 21 02 116 120 10.1080/10401330902791099 19330689

[10] Schubart J R Erdahl L Smith J S Jr Purichia H Kauffman G L Kass R B Use of breast simulators compared with standardized patients in teaching the clinical breast examination to medical students J Surg Educ 2012 69 03 416 422 10.1016/j.jsurg.2011.10.005 22483147

[11] Association for Surgical Education Pugh C M Salud L H Fear of missing a lesion: use of simulated breast models to decrease student anxiety when learning clinical breast examinations Am J Surg 2007 193 06 766 770 10.1016/j.amjsurg.2006.12.033 17512293

[12] Siwe K Wijma K Validation of the Fear of Pelvic Examination Scale (F-PEXS)–measuring students' fear of performing a pelvic examination J Psychosom Obstet Gynaecol 2015 36 01 23 28 10.3109/0167482X.2014.994500 25541215

[13] Abraham S Chapman M Taylor A McBride A Boyd C Anxiety and feelings of medical students conducting their first gynecological examination J Psychosom Obstet Gynaecol 2003 24 01 39 44 10.3109/01674820309042799 12685338

[14] Deladisma A M Gupta M Kotranza A Bittner J G IV A pilot study to integrate an immersive virtual patient with a breast complaint and breast examination simulator into a surgery clerkship Am J Surg 2009 197 01 102 106 10.1016/j.amjsurg.2008.08.012 19101251

